# Vascular Lesions on the Left Upper Gingiva in a Patient With Port‐Wine Stains

**DOI:** 10.1002/ccr3.70707

**Published:** 2025-08-11

**Authors:** Tinglan Yang, Mengmeng Song, Qing Liu, Zhenlai Zhu

**Affiliations:** ^1^ State Key Laboratory of Oral & Maxillofacial Reconstruction and Regeneration, National Clinical Research Center for Oral Diseases, Shaanxi Key Laboratory of Stomatology, Department of Oral Medicine School of Stomatology, The Fourth Military Medical University Xi'an Shaanxi P. R. China

**Keywords:** capillary hemangioma, patient care team, photodynamic therapy, port‐wine stain, vascular malformation

## Abstract

Port‐wine stain patients may develop asymptomatic intraoral capillary hemangiomas ipsilateral to facial lesions. Multidisciplinary management is critical to differentiating these from neoplasms, mitigating bleeding risks, and optimizing therapies like photodynamic treatment. Regular oral monitoring is advised to detect vascular proliferation and dental abnormalities.

## 1 Case Presentation

**FIGURE 1:**
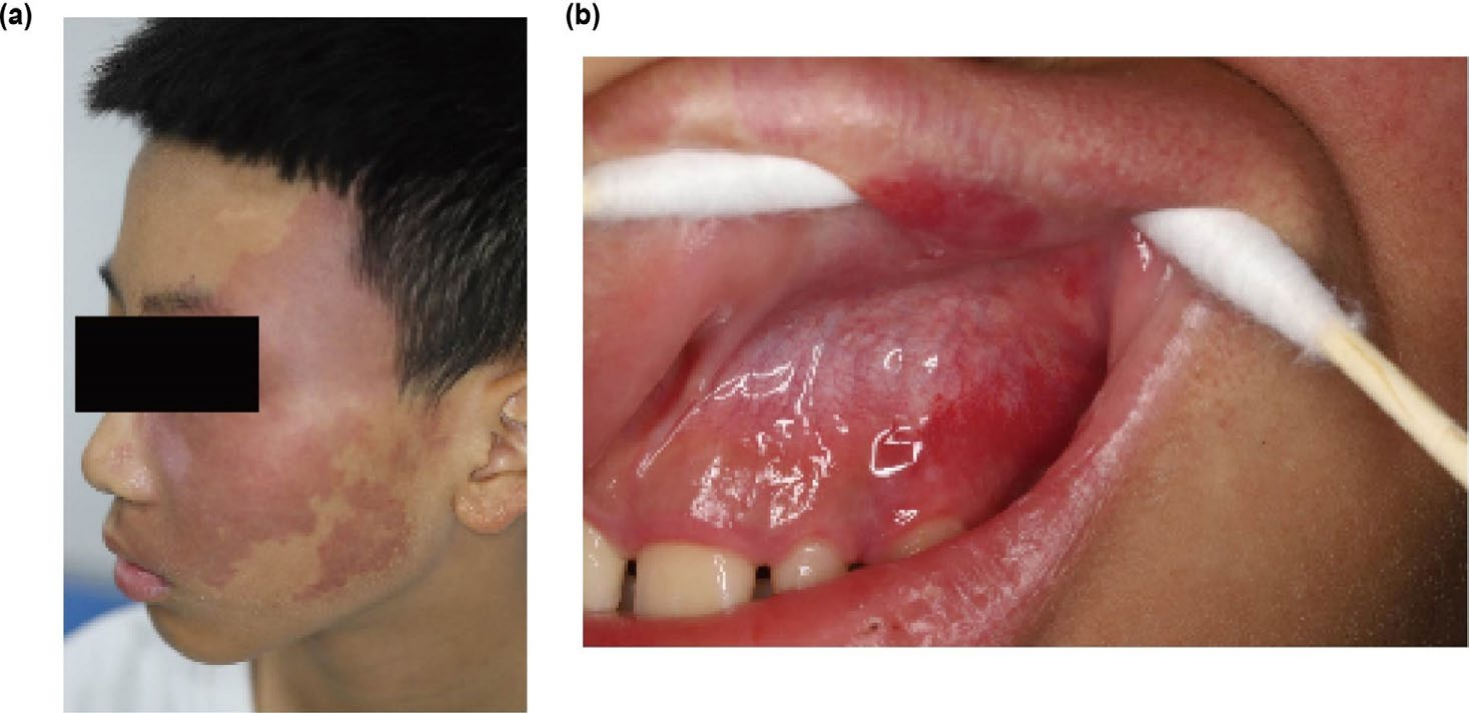
Clinical images. (a) Port‐wine stain along the distribution of the left maxillary nerve. (b) Lesions on the maxillary left gingiva, alveolar, and labial mucosa.

A 13‐year‐old boy with a congenital port‐wine stain (PWS) on the left facial region (Figure 1a) had undergone multiple photodynamic therapy sessions over the past year, achieving significant erythema reduction. Two weeks prior to presentation, his parents noticed asymptomatic red lesions on his maxillary left gingiva (Figure 1b). The patient presented with no history of systemic diseases, seizures, or visual disturbances. Physical examination revealed an erythematous patch distributed along the left maxillary branch of the trigeminal nerve, with an irregular shape and well‐defined borders. Intraoral examination revealed bright fiery‐red areas on the maxillary left buccal gingiva, palatal gingiva, alveolar mucosa, and labial mucosa. The masticatory mucosa of the hard palate and soft palate mucosa were unaffected. Intraoral diascopy was performed, revealing blanching of the lesions. No systemic symptoms, bleeding tendency, or contralateral involvement were noted. The differential diagnoses considered included pyogenic granuloma and venous malformations. Pyogenic granuloma was deemed unlikely given the lack of bleeding tendency or trauma history, while venous malformations were ruled out based on the non‐purplish color and capillary morphology of the lesions. Following oral health education, the patient continued with photodynamic therapy for facial and oral lesions with the involvement of oral medicine specialists. The patient is currently under follow‐up with no adverse outcomes reported.

## 2 Discussion

PWSs are congenital vascular malformations that appear clinically as erythematous areas on the buccal mucosa, vermilion border of the lip, gingiva, or as pink to port‐wine‐colored patches on the skin. The intraoral capillary hemangiomas, observed in 40% of patients, are often overlooked due to their asymptomatic nature [1]. Regular oral examinations are critical for early detection of vascular proliferations and dental anomalies, such as malocclusion, associated with angiomatosis [2]. Our patient showed no features of syndromic PWS associations, specifically lacking both the neuro‐ophthalmic abnormalities characteristic of Sturge–Weber syndrome and the limb hypertrophy or lymphatic malformations of Klippel–Trenaunay–Weber syndrome. In some cases, there may be further proliferation on the basis of capillary hemangioma, leading to the formation of lobular capillary hemangioma [3]. Consequently, the involved mucosa becomes delicate and susceptible to even minor injuries and simple dental operations. It is noteworthy that most PWS patients do not have coagulation abnormalities but tend to bleed more easily during treatments. Additionally, recent studies have reported abnormal tooth maturation on the affected side in PWS patients; thus, attention should be paid to the tooth maturation on the affected side during oral examinations [2]. This case underscores the necessity of a multidisciplinary approach in managing PWS to ensure comprehensive care and achieve optimal outcomes.

## Author Contributions

**Tinglan Yang:** writing – original draft, writing – review and editing. **Mengmeng Song:** data curation, investigation. **Qing Liu:** data curation, project administration, resources, supervision. **Zhenlai Zhu:** data curation, investigation, writing – original draft, writing – review and editing.

## Consent

We have obtained the written informed consent from the patient's parents.

## Conflicts of Interest

The authors declare no conflicts of interest.

## Data Availability

The data that support the findings of this study are available from the corresponding author upon reasonable request.
